# Ultrasonographic manifestations and misdiagnosis analysis of ovarian leiomyoma

**DOI:** 10.3389/fmed.2023.1289522

**Published:** 2023-12-27

**Authors:** Min Zhang, Yan Hong, Li Wang, Ling-Ling Qin, Xiang-xiang Jing

**Affiliations:** Department of Ultrasonography, Hainan General Hospital/Hainan Affiliated Hospital of Hainan Medical University, Haikou, Hainan, China

**Keywords:** ovarian leiomyoma, solid tumor, brief research report, diagnosis, ultrasonographic manifestations

## Abstract

**Objectives:**

Ovarian leiomyomas (OLs) are rare and account for only 0.5 to 1% of benign ovarian tumors. This study investigated the ultrasonographic manifestations of OL and the potential reasons for misdiagnosis.

**Methods:**

Between July 2018 and July 2023, 7 patients diagnosed with OL by surgical pathology and immunohistochemistry were enrolled in this retrospective analysis. Ultrasound (US) examinations were performed before surgery. Clinical characteristics, pathological findings, ultrasonographic manifestations, and treatment were reviewed.

**Results:**

The mean age of the 7 patients was 39.0 ± 11.57 years, with a disease course of 0.1 to 24 months. All ovarian leiomyomas were unilateral. Four cases occurred in the right ovary, and three cases occurred in the left ovary. All lesions presented as hypoechogenic, well-circumscribed, round or oval in shape, and regular in morphology. No significant blood flow signal was detected peripheral to or inside the mass in 3 cases (42.8%), and a minimal flow signal was detected peripheral to or inside the mass in 4 cases (58.2%). A total of 7 ultrasonographic images of OL were misdiagnosed: 1 patient was misdiagnosed with subserosal uterine leiomyoma, and 6 patients were misdiagnosed with a tumor in the ovarian thecoma–fibroma group.

**Conclusion:**

The imaging manifestation of OL lacks specificity; thus, preoperatively distinguishing OL from other ovarian tumors and subserosal uterine leiomyomas is difficult. Immunohistochemistry may be helpful for the definitive diagnosis of OL. The possibility of ovarian leiomyoma should be considered in patients with uterine leiomyomas coexisting with an adnexal ovarian solid mass.

## 1 Introduction

Ovarian leiomyomas were first described by Sangalli in 1862, accounting for 0.5–1% of benign ovarian tumors (1). Fewer than 200 cases have been reported thus far, most of which were presented in case reports or case series. Clinically, OL is asymptomatic and detected incidentally during medical check-ups (2). Typically, ovarian leiomyoma is unilateral with no side predilection and occurs mostly in premenopausal patients (3). Given the low clinical incidence and difficulty in distinguishing it from other ovarian tumors and subserosal leiomyoma, OL is seldom diagnosed before surgery. Therefore, histopathological examination and immunohistochemistry are the common methods used to clarify the diagnosis of OL (4). In this study, a retrospective review of clinical information, ultrasonographic manifestations, and the reasons for misdiagnosis for seven patients diagnosed with OL was performed.

## 2 Patients and equipment

Seven patients diagnosed with OL by surgical pathology and immunohistochemistry in the Hainan General Hospital from July 2018 to July 2023 were enrolled in this retrospective analysis. A HITACHI VISION Ascendus scanner (Hitachi Manufacturing Co., Ltd., Chiba Prefecture, Japan) and a Voluson E8 (GE Healthcare, USA), which is equipped with a vaginal probe (5–9 MHz) and a C4-8-D convex-array transducer (4–8 MHz), were used for ultrasound (US) examinations in this study. Patients’ clinical data were obtained from electronic patient records. The preoperative diagnosis in this study depended on ultrasound findings. Ethics committee approval was obtained from the Medical Ethics Committee of Hainan General Hospital (Ethics Approval No.: Med-Eth-Re [2023] 324). Written informed consent was obtained from each participant.

## 3 Clinical features and ultrasonographic manifestations of seven ovarian leiomyomas

The mean age of the 7 patients was 39.0 ± 11.57 years, with disease duration of 0.1 to 24 months, and the average course was 7.02 ± 8.63 months. The admission complaint of 5 (71.4%) patients was an asymptomatic pelvic mass. One patient (14.3%) complained of right abdominal pain, and the other patient (14.3%) complained of irregular vaginal bleeding. All ovarian leiomyomas were unilateral; the leiomyomas were located in the left ovary in 3 (42.8%) cases and in the right ovary in 4 (57.2%) cases. The estrogen levels were normal in all cases. The CA125 level was normal in 6 (85.7%) out of 7 cases but slightly increased in 1 (14.2%) out of 7 cases.

**Figure 1:**
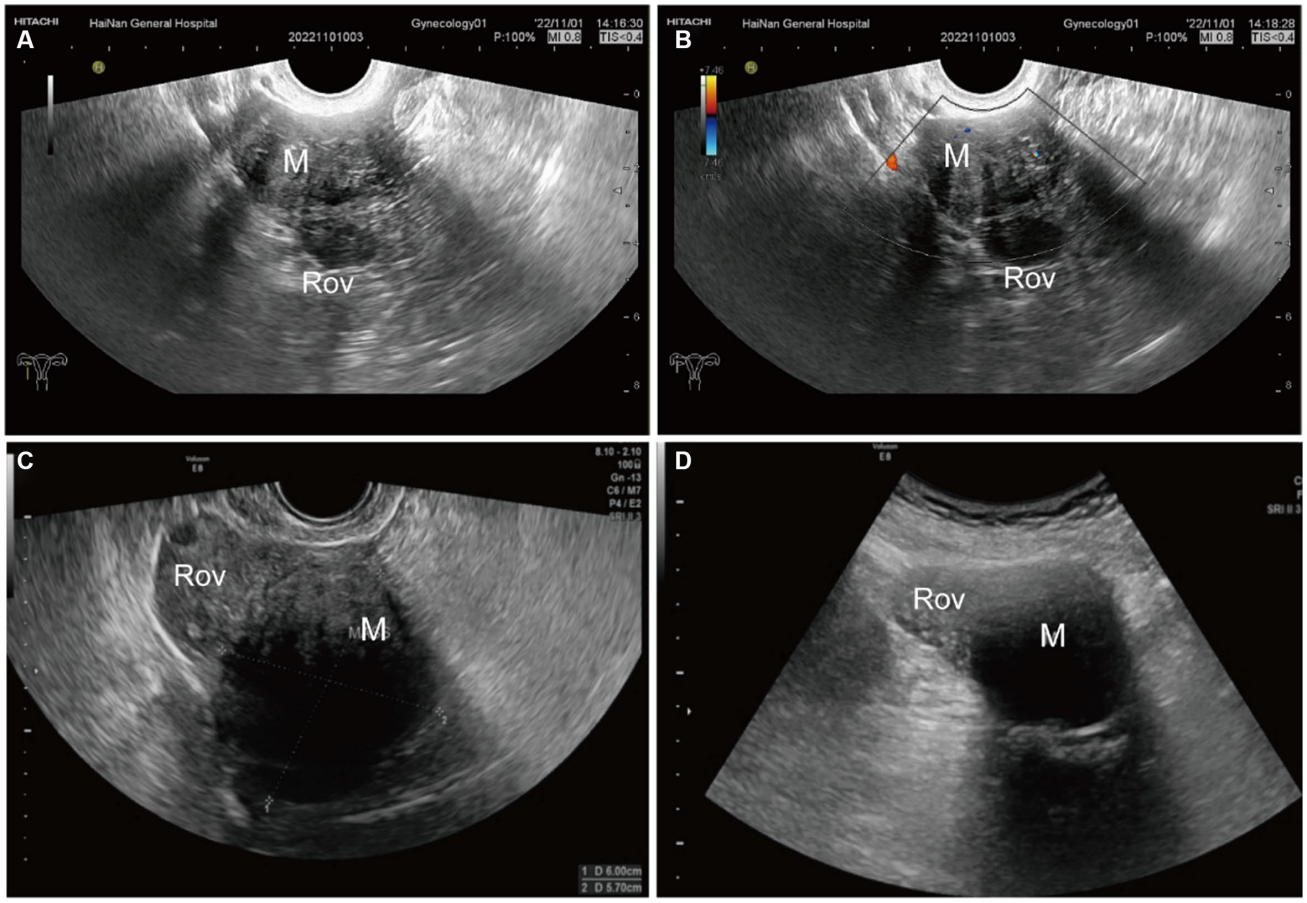
Ultrasonographic imaging of OL localized to the ovary periphery in case 6 **(A,B)** and case 5 **(C,D)**. **(A)** A heterogeneous hypoechogenic, well-circumscribed, oval-shaped adnexal mass adjacent to the right ovary was detected by transvaginal ultrasound. The mass size was 42 mm × 29 mm. **(B)** Punctate blood flow signals around the mass were detected by color Doppler ultrasound. **(C,D)** Transvaginal ultrasound **(C)** and transabdominal ultrasound imaging **(D)** demonstrated a heterogeneous mass with more hypoechoic signal than the surrounding ovary and echo attenuation. The mass size was 69 mm × 53 mm. ROV, right ovary; M, mass.

**Figure 2:**
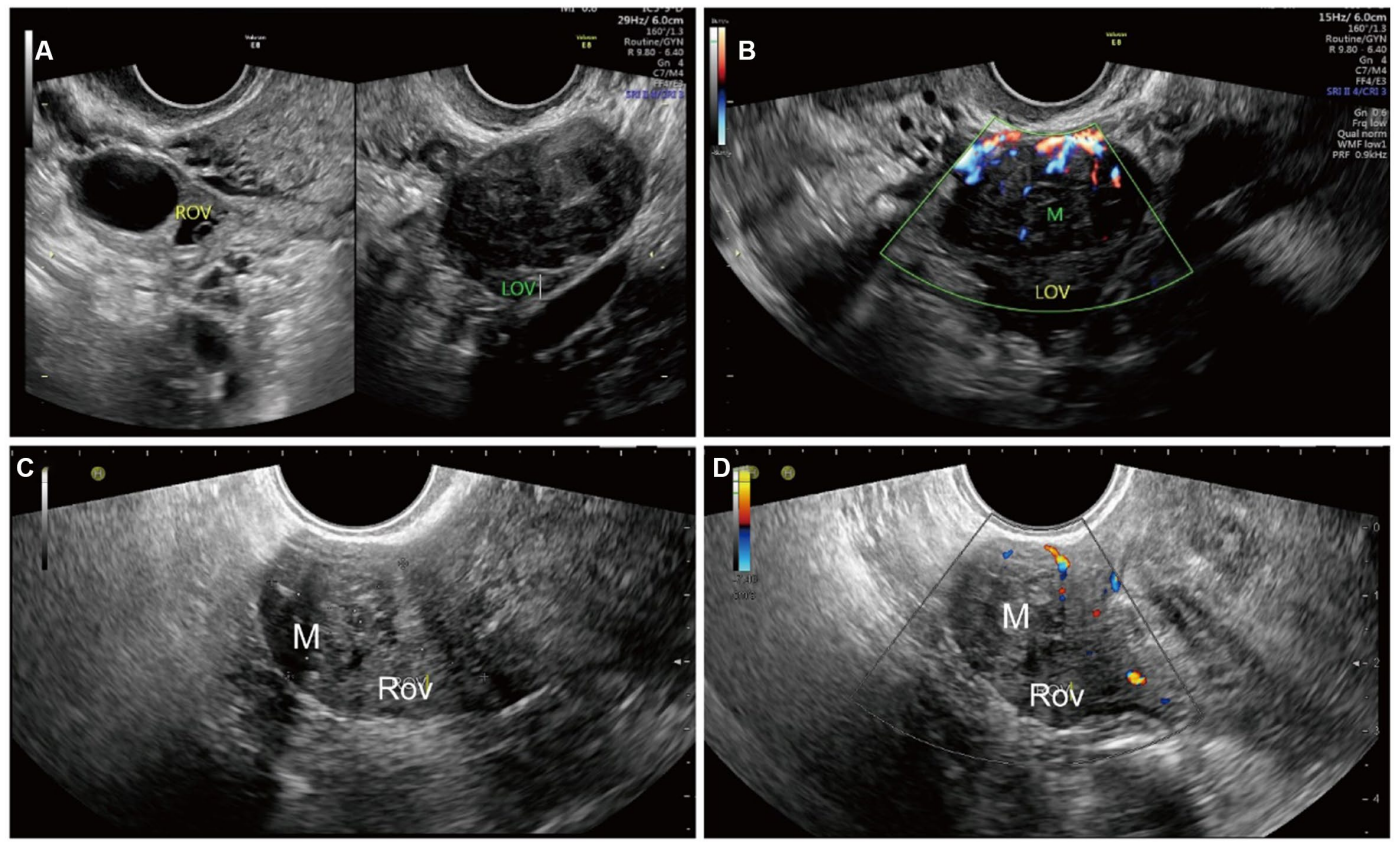
Ultrasonographic imaging of an OL localized inside the ovary in case 4 **(A,B)** and case 2 **(C,D)**. **(A)** A well-circumscribed, heterogeneous ovarian mass was detected by transvaginal ultrasound. The mass was hypoechogenic and more hypoechoic than the surrounding ovary. **(B)** Strip-color flow signals in and around the mass were detected by color Doppler ultrasound. **(C)** A well-circumscribed, heterogeneous hypoechogenic ovarian mass with echogenicity equal to that of the surroundings was detected by transvaginal ultrasound. The mass was measured to be 19 mm × 17 mm. **(D)** Strip-color flow signals in and around the mass were detected by color Doppler ultrasound. The patient was treated with laparoscopic mass excision. The patient is alive without postoperative complications or tumor recurrence 1 year after surgery. ROV, right ovary; M, mass.

The largest diameter measured using ultrasound was 7.0 cm, and the smallest diameter was 1.9 cm. The lesions were localized to the ovary periphery in two cases (Figure 1) and inside the ovary in five cases (Figure 2). All OL lesions presented as heterogeneous hypoechogenic, well-circumscribed, round or oval in shape, and regular in morphology. There were no foci of liquefaction or calcification in the mass. Echo attenuation behind the mass was detected in 2 cases (28.6%) by ultrasound. No significant blood flow signal was detected peripheral to or inside the mass in 3 cases (42.8%), and a minimal flow signal was detected peripheral to or inside the mass in 4 cases (58.2%). Among the 7 enrolled patients, 3 (42.8%) had concomitant uterine leiomyomas, 2 (28.6%) had concomitant endometrial polyps, and 1 (14.3%) had ascites. Of the 7 patients, 6 patients underwent adnexectomy, and the remaining 1 patient underwent mass excision.

**Figure 3:**
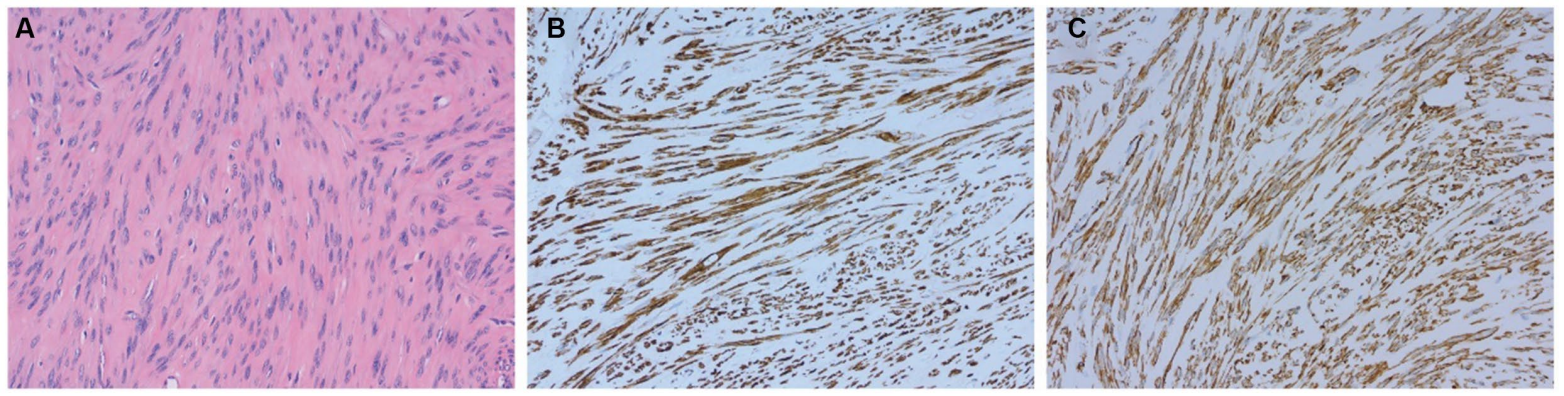
Pathological results confirming the diagnosis of ovarian leiomyoma in case 4. **(A)** Hematoxylin and eosin (H-E) staining of the OL (magnification × 200). **(B,C)** Expression of smooth muscle markers, including desmin **(B)** and α-smooth muscle actin **(C)**, as determined by immunohistochemical analyses (magnification × 200).

Hematoxylin and eosin (H-E) staining showed that in all OL cases, the OLs were composed of uniform long spindle-shaped cells without obvious nuclear atypia and with eosinophilic cytoplasm. Positive α-smooth muscle actin and desmin expression were determined by immunohistochemical analyses in most cases, confirming the diagnosis of ovarian leiomyoma (Figure 3).

Postoperative recurrence was evaluated by transvaginal ultrasound during follow-up. All patients without postoperative therapy were followed up by hospital visits or phone calls. The follow-up period ranged from 7 to 55 months (mean period, 22.5 months). None of the patients showed evidence of tumor recurrence.

A total of 7 ultrasonographic images of OL were misdiagnosed, whereby 1 case was misdiagnosed as subserosal uterine leiomyoma and 6 cases were misdiagnosed as ovarian thecoma–fibroma. Clinical and ultrasonographic data are summarized in Table 1.

**Table 1:** Clinical and ultrasonographic features of seven OL patients.

Case	Age (years)	Tumor Size (mm × mm)	contralateral uterus and ovary	Preoperative ultrasonographic diagnosis	CA125 (IU/mL) Normal value <35 IU/mL	Treatment	Follow-up period (months)	Immunohistochemical staining		
								SMA	desmin	α-inhibition
1	18	70 × 60	/	Ovarian thecoma–fibroma group	17.2	Abdominal adnexectomy	14	(+)	(+)	(−)
2	43	19 × 17	Endometrial polyps and uterine leiomyomas	Ovarian thecoma–fibroma group	15.4	Laparoscopic mass excision	13	(+)	(+)	/
3	48	23 × 17	Uterine leiomyomas	Ovarian tumor	7.05	Laparoscopic adnexectomy	27	/	/	/
4	42	37 × 25	Uterine leiomyomas	Ovarian thecoma–fibroma group	10.6	Laparoscopic mass excision	12	(+)	(+)	(−)
5	28	66 × 53	A small amount of ascites	Ovarian thecoma–fibroma group	71.7	Abdominal adnexectomy	30	(+)	(+)	/
6	45	42 × 29	Endometrial polyps	Subserosal uterine leiomyomas	5.2	Laparoscopic adnexectomy	7	(+)	(+)	(−)
7	49	69 × 53	/	Ovarian thecoma–fibroma group	23.9	Abdominal adnexectomy	55	(+)	(+)	/

## 4 Discussion

The origin of OL is still somewhat controversial. Some scholars believe that OL may arise from the ovarian ligament, smooth muscle cells, and blood vessels of the ovarian hilar, while other scholars believe that OL probably originates from undifferentiated germ stem cells of the ovarian stroma. Another study confirmed that OL originates from ovarian stromal cell metaplasia into smooth muscle cells (5).

The coexistence of ovarian leiomyomas with uterine leiomyomas has been reported (6, 7). In this group of 7 OL patients, 3 had uterine leiomyomas and 2 had endometrial polyps, which are in good accordance with reports in the literature. Similar to uterine leiomyoma, estrogen promotes the growth of OL. Because the level of estrogen decreases after menopause, the incidence is significantly decreased in postmenopausal women (8, 9).

The ultrasonographic manifestations of OL presented as typical benign ovarian tumors, including heterogeneous hypoechogenic, well-circumscribed, round, oval in shape, and no significant blood flow signal or minimal flow signal within the mass in the present study. None of the patients were properly diagnosed before the operation, 6 patients were misdiagnosed as having a tumor in the ovarian thecoma–fibroma group, and another patient was misdiagnosed as having subserosal uterine leiomyomas. The definitive diagnosis of OL depends mainly on pathology and immunohistochemistry. The differential diagnosis of OL includes subserosal uterine leiomyomas, broad ligament leiomyomas, diffuse peritoneal leiomyomatosis, intravascular leiomyomatosis, and ovarian thecoma–fibroma groups. Furthermore, OL with cystic degeneration or calcification also needs to be differentiated from ovarian cysts with extensive mural fibrosis (10–12). Careful detection, abdominal compression, and position changes could facilitate US in the evaluation of the relationship between the mass and the uterus. The causes of misdiagnosis may be attributed to the rarity and non-specific imaging appearance of OL as well as the insufficient clinical experience of radiologists.

OL is a rare benign tumor without a tendency toward recurrence and malignant change. Regarding OL, adnexectomy on the affected side is necessary for most patients. In principle, for patients who have fertility requirements, mass excision is a better option. Age, tumor size, and fertility preservation should be considered in therapy selection. All OL patients had a good prognosis.

The study still suffers from the limitations of a small number of patients and low prevalence rates. Further studies in clinical settings need to be performed.

## 5 Conclusion

The clinical and imaging manifestations of OT lack specificity, resulting in difficulty in preoperative diagnosis. Ovarian leiomyoma should be considered in patients with a heterogeneous hypoechogenic, well-circumscribed, oval-shaped adnexal mass, coexisting with uterine leiomyomas. The tumor markers and estrogen levels are usually normal, and immunohistochemistry may be helpful for the definitive diagnosis of OL.

## Data availability statement

The raw data supporting the conclusions of this article will be made available by the authors, without undue reservation.

## Ethics statement

Written informed consent was obtained from the individual(s) for the publication of any potentially identifiable images or data included in this article.

## Author contributions

MZ: Writing – original draft. YH: Data curation, Writing – review & editing. LW: Data curation, Writing – original draft. L-LQ: Project administration, Writing – review & editing. X-xJ: Supervision, Writing – review & editing.
